# The co-administration of live fowlpox and Newcastle disease vaccines by non-invasive routes to chickens reared by smallholders in Tanzania and Nepal

**DOI:** 10.1007/s11250-022-03250-3

**Published:** 2022-09-23

**Authors:** Stuart J. Andrews, Asanteli Makundi, Julius Mwanadota, Sakar Shivakoti, Bikas Raj Shah, E. Jane Poole, Jeremy Salt, Kristin Stuke

**Affiliations:** 1 Canterbury, UK; 2 Faculty of Science and Environmental Studies, The Open University of Tanzania, P.O. Box 271, Babati, Tanzania; 3 Centre for Infectious Diseases of the Tanzania Veterinary Laboratory Agency (TVLA), Dar-es-Salaam, Tanzania; 4 Heifer International Nepal, Ward No. 15, Lalitpur Metropolitan, Nepal; 5 Statistics for Sustainable Development (Stats4SD), 6, Southern Court, South Street, Reading, RG1 4QS UK; 6 GALVmed UK Office, Doherty Building, Pentlands Science Park, Bush Loan, Penicuik, Edinburgh EH26 0PZ UK; 7 Global Alliance for Livestock Veterinary Medicines (GALVmed), Nairobi, Kenya

**Keywords:** Newcastle disease, Fowlpox, Vaccination, Feather follicle, Eye-drop, Chickens

## Abstract

The co-administration of commercial live fowlpox (FP) and Newcastle disease (ND) vaccines when given by non-invasive (needle-free) routes was demonstrated to be safe and to elicit immunity in two field studies, one in Tanzania the other in Nepal. Both studies were of a cluster-randomised controlled design in which birds were randomly assigned to one of five treatment groups: (i) administration with FP vaccine alone (feather follicle), (ii) administration with ND vaccine alone (eye-drop), (iii) concurrent administration of FP (feather follicle) and ND (eye-drop) vaccines, (iv) concurrent administration of FP (wing-web) and ND (eye-drop) vaccines, and (v) unvaccinated, acting as environmental sentinels. Data from a total of 1167 birds from seven villages in Hanang District of Tanzania together with 1037 birds from eleven villages in Dhading District of Nepal were collected over a period of 21 and 28 days, respectively. Immune responses to FP vaccination were evaluated by local take reactions, while those to ND vaccination were evaluated serologically by haemagglutination inhibition test. The two studies demonstrated that the concurrent vaccination of free-range, indigenous breeds of chicken with live FP and ND vaccines, both administered by non-invasive routes, was safe and induced immunity against FP and ND that were non-inferior to the administration of FP and ND vaccines alone. These findings are important to appropriately trained small-scale backyard poultry farmers as well as to paraprofessionals and community health workers helping to increase vaccine uptake and the control of both FP and ND in low- to middle-income countries.

## Introduction

Fowlpox (FP) and Newcastle disease (ND) continue to be major constraints on the production of poultry, especially chickens, in the rural communities of many low- to middle-income countries (LMICs), causing mortality, poor growth rate, and drop in egg production (Alexander 2001; Tripathy and Reed 2008; Campbell et al 2021). Commercial live vaccines, including FP-ND combination and vectored FP-ND combination vaccines are licensed to help prevent and control both FP and ND; however, the multi-valent and vectored combination vaccines are generally not available or are inappropriate for use (large dose size, inconvenient route of administration, cold chain management) in the rural backyard setting of most LMICs. Commercial live monovalent FP and ND vaccines are, however, generally available in the local markets of most LMICs. Currently, ND vaccines can be administered by eye-drop, a non-invasive route, by trained paraprofessionals and community health workers (Alders and Spradbrow 2001). There is an opportunity for these workers, and indeed in some rural areas also appropriately trained farmers, to administer live FP vaccines by feather follicle, also a non-invasive route, at the same time. Such co-administration of FP and ND vaccines would be beneficial in terms of reducing the number of separate interventions and therefore cost.

The results of a proof of concept (POC) study carried out by GALVmed showed that the majority of chickens that were vaccinated against FP by the feather follicle route were immunised, as shown by the development of take reactions at the site of vaccine administration. All of the birds that were vaccinated against ND by eye-drop developed good levels of protective antibody as shown by serology. Immune interference due to the co-administration of both FP and ND vaccines was not observed (Stuke et al 2017). This paper presents the results of two field studies designed to confirm the findings of the POC study and to demonstrate that the co-administration of commercial live FP and ND vaccines, when both are given by non-invasive routes, is safe and elicits protective immunity. The first study was carried out in Tanzania between August to September 2018 in partnership with the Open University in Tanzania and the second in Nepal between January to March 2021 in partnership with Heifer International Nepal, both using free-range, scavenging, indigenous chickens representative of the local populations, in the rural backyard and village setting.

## Materials and methods

### Study area, village, and household selection

#### Tanzania

This study was carried out in seven villages: Bassotu, Bassotughang, Dawari, Gehandu, Lalaji, Simbay, and Wareta located within the Hanang District of the Manyara region of Tanzania. An average of 7 households to each village and group, 49 households (237 birds) were assigned to group 1, 48 households (233 birds) to group 2, 51 households (238 birds) to group 3, 45 households (218 birds) to group 4, and 49 households (241 birds) to group 5.

#### Nepal

This study was carried out in eleven villages: Ashare, Batase, Bhirpani, Chainpur, Dadagau, Deurali, Kattlepauwa, Majhigau, Melewar, Simpani, and Tallo D located within the Dhading District of the Bagmati Province of Nepal. For formal statistical analysis, the eleven villages were grouped into nine spatial clusters. An average of 5 households were assigned to each spatial cluster and group, with 223 birds in group 1, 200 birds in group 2, 199 birds in group 3, 208 birds in group 4, and 207 birds in group 5.

All villages, in both studies, were selected based on the ease of access, an adequate number of chickens per household to meet the minimum number required to provide sufficient statistical power to support non-inferiority statements with respect to FP vaccine take reaction rates and ND serology at the end of the study, and having no history of vaccination against either FP or ND. Relevant information was obtained by visiting each household prior to the start of the study to assess their willingness to participate in the study and to obtain baseline data using a standardised questionnaire. Each owner signed an owner informed consent form, and legal and ethical approval was obtained from the appropriate government department: Ministry of Agriculture Livestock and Fisheries of the Republic of Tanzania, and the Social Welfare and Veterinary Councils of Nepal.

### Study design

The design of each field study was similar and followed a cluster-randomised controlled study design the key features of which are shown in Tables 1 and 2.

**Table 1:** Design of the study carried out in Tanzania

Group	Number of birds	Number of households	Vaccine	Dose volume per vaccination (mL)	Route of vaccination	Age at vaccination (weeks)
1	237	49	FP	0.017*	Feather follicle	4–16
2	233	48	ND	0.05	Eye-drop	4–16
3	238	51	ND + FP	0.05 + 0.017*	Eye-drop + Feather follicle	4–16
4	218	45	ND + FP	0.05 + 0.01	Eye-drop + Wing-web	4–16
5	241	49	n/a	n/a	n/a	n/a

*FP* fowlpox vaccine, *ND* Newcastle disease vaccine, *n/a* not applicable

*The dose volume of FP vaccine used by feather follicle administration was determined in the POC study (Stuke et al, 2017)

**Table 2:** Design of the study carried out in Nepal

Group	Number of birds	Number of households	Vaccine	Dose volume per vaccination (mL)	Route of vaccination	Age at vaccination (weeks)
1	223	45	FP	0.017*	Feather follicle	6–18
2	200	43	ND	0.03	Eye-drop	6–18
3	199	44	ND + FP	0.03 + 0.017*	Eye-drop + Feather follicle	6–18
4	208	44	ND + FP	0.03 + 0.006**	Eye-drop + Wing-web	6–18
5	207	45	n/a	n/a	n/a	n/a

*FP* fowlpox vaccine, *ND* Newcastle disease vaccine, *n/a* not applicable

*The dose volume of FP vaccine used by feather follicle administration was determined in the POC study (Stuke et al, 2017).

**Dose volume of double needle as indicated by the vaccine manufacturer

After completion of the site survey, each household in each village was randomly allocated to one of five treatment groups: (i) administration with FP vaccine alone (feather follicle), (ii) administration of ND vaccine alone (eye-drop), (iii) concurrent administration of FP (feather follicle) and ND (eye-drop) vaccines, (iv) concurrent administration of FP (wing-web) and ND (eye-drop) vaccines, and (v) unvaccinated, acting as environmental sentinels. Just prior to vaccination (day 0), the general health of each bird was checked before being given a unique ID. The birds in groups 1–4 were then vaccinated, those in group 5 acting as unvaccinated environmental sentinels. Chickens were at least 4–6 weeks old at the time of vaccination in order to meet the vaccine manufacturer’s product label indications which in doing so would reduce the likelihood of previous exposure to FP and ND viruses, avoid age-related mortality in very young birds, and ensure that maternally derived antibodies had waned so not neutralising the effect of the vaccines. Chickens were not older than 16–18 weeks at the time of vaccination to try and reduce the risk of them being sold or consumed alongside adult birds, and to exclude birds in or just before the onset of the laying period as FP vaccines are known to cause a drop in egg production if birds are vaccinated during the laying period.

On the day of vaccination, blood samples were collected to measure antibodies against ND virus using a haemagglutination inhibition (HI) test: La Sota antigen, 4 haemagglutination units (HAU) of antigen. On three other separate occasions: 7, 9, and 21 days after vaccination in Tanzania and 7, 11, and 28 days after vaccination in Nepal, the administration sites of the FP vaccination of birds in groups 1, 3, and 4 were assessed for the development of takes. A take consists of a nodule, swelling, or scab on the skin at the site where the vaccine was applied and its presence 7–10 days after vaccination is evidence of successful immunisation. The personnel assessing the take reactions were not blinded to treatment group. The studies ended either 21 days (Tanzania) or 28 days (Nepal) after vaccination, the differences in study duration being due to logistical reasons.

All data summaries and statistical analysis was performed using the R^©^ statistical computing platform.

### Vaccines

#### Newcastle disease vaccine

Tanzania—CEVAC NEW L® (Ceva). Live vaccine containing the La Sota lentogenic strain of ND virus of chicken embryo origin. Each 0.03 mL dose contained at least 10^5.5^ EID_50_ of live attenuated ND virus. Before vaccination by eye-drop, each 500-dose vial was dissolved with 30 mL of sterile diluent and then sub-divided into 10 mL plastic droppers with an estimated volume of 0.05 mL per drop* [*a mistake in the field with diluting the vaccine was rectified by increasing the volume of each dose of vaccine to 0.05 mL per drop].

Nepal—LIVE LAS® (Hester Biosciences Nepal Private Limited). Live vaccine containing the La Sota lentogenic strain of ND virus of chicken embryo origin. Each 0.03 mL dose contained at least 10^7.35^ EID_50_ of live attenuated ND virus. Before vaccination by eye-drop, each 200-dose vial was rehydrated with one vial containing 6 mL of vaccine diluent.

#### Fowlpox vaccine

**Fig. 1:**
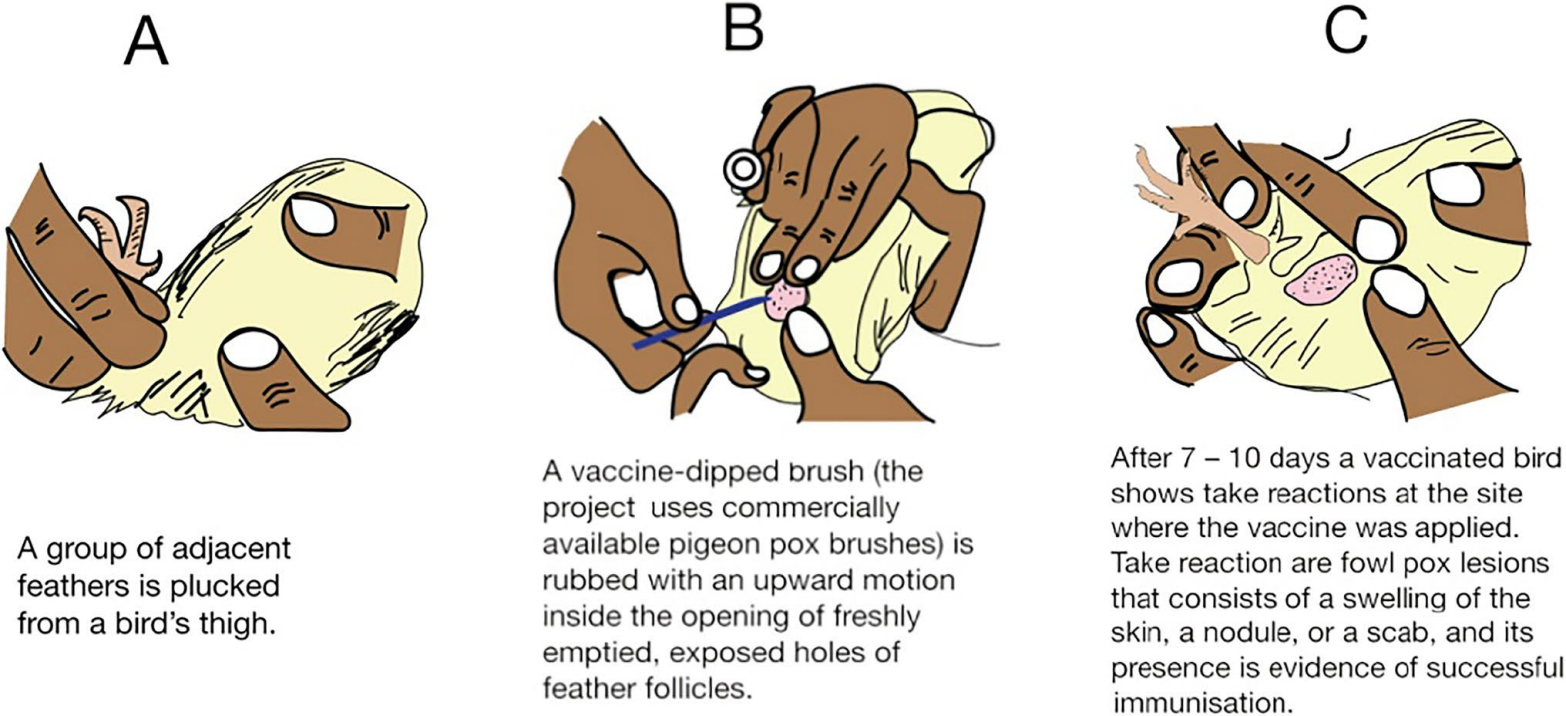
Artwork designed to show the application of live fowlpox vaccine using the feather follicle technique

Tanzania—CEVAC FP L® (Ceva). Live vaccine containing the PII (Cutter) strain of fowlpox virus of fowl embryo origin. Each dose of 0.017 mL (as determined by the POC study (Stuke et al 2017)) contained at least 10^2.2^ EID_50_ of live attenuated fowlpox virus. Before vaccination, each 1000-dose vial was dissolved with 10 mL of sterile diluent. For the feather follicle vaccination method, a group of adjacent feathers from an area of about 0.5–1.0 cm^2^ on a bird’s thigh was plucked and a vaccine-dipped brush (a commercially available pigeon pox microtip applicator was used) rubbed with an upward motion inside the opening of the exposed feather follicle. Disinfectant was not used on the area of application as this would have inactivated the vaccine. A new brush was used for every household/flock. For the wing-web vaccination method, a two-pronged needle applicator was dipped into the vaccine until it filled the grooves of the stabber with 0.01 mL per dose and a featherless spot on the underside of the centre of the wing-web was penetrated, avoiding feathers, blood vessels, and bones (Fig. 1).

Nepal—FOWL POX VACCINE (Indovax, India). Live vaccine containing FP virus (the identity of the strain was not disclosed by the manufacturer) of specified pathogen free chick embryo origin. Each 0.006 mL dose formulated to contain at least 10^2^ EID_50_ of live attenuated FP virus. Each 500-dose vial was rehydrated with one 3 mL vial of FP vaccine diluent. For the feather follicle vaccination method, a group of adjacent feathers from an area of about 0.5–1.0 cm^2^ on a bird’s thigh was plucked and a vaccine-dipped brush rubbed with an upward motion inside the opening of the exposed feather follicle. Disinfectant was not used on the area of application as this would have inactivated the vaccine. A new brush was used for every household/flock. For the wing-web vaccination method, a two-pronged needle applicator was dipped into the vaccine until it filled the grooves of the stabber with 0.006 mL per dose and a featherless spot on the underside of the centre of the wing-web was penetrated, avoiding feathers, blood vessels, and bones.

## Results

### Field study in Tanzania

#### Study population

The study population consisted of 56% (657/1167) female and 42% (490/1167) male birds (data for 2% (20/1167) of the birds were not available), with birds of different sex and age being represented in all groups.

### Mortality

A total of 63 birds died during the study: 9/217 in group 1, 6/206 in group 2, 8/222 in group 3, 21/202 in group 4, and 19/225 in group 5. Predator attack was reported as the main cause of mortality (range 33–75%). No adverse events were reported to be attributable to either ND or FP vaccination.

#### Take reactions

**Fig. 2:**
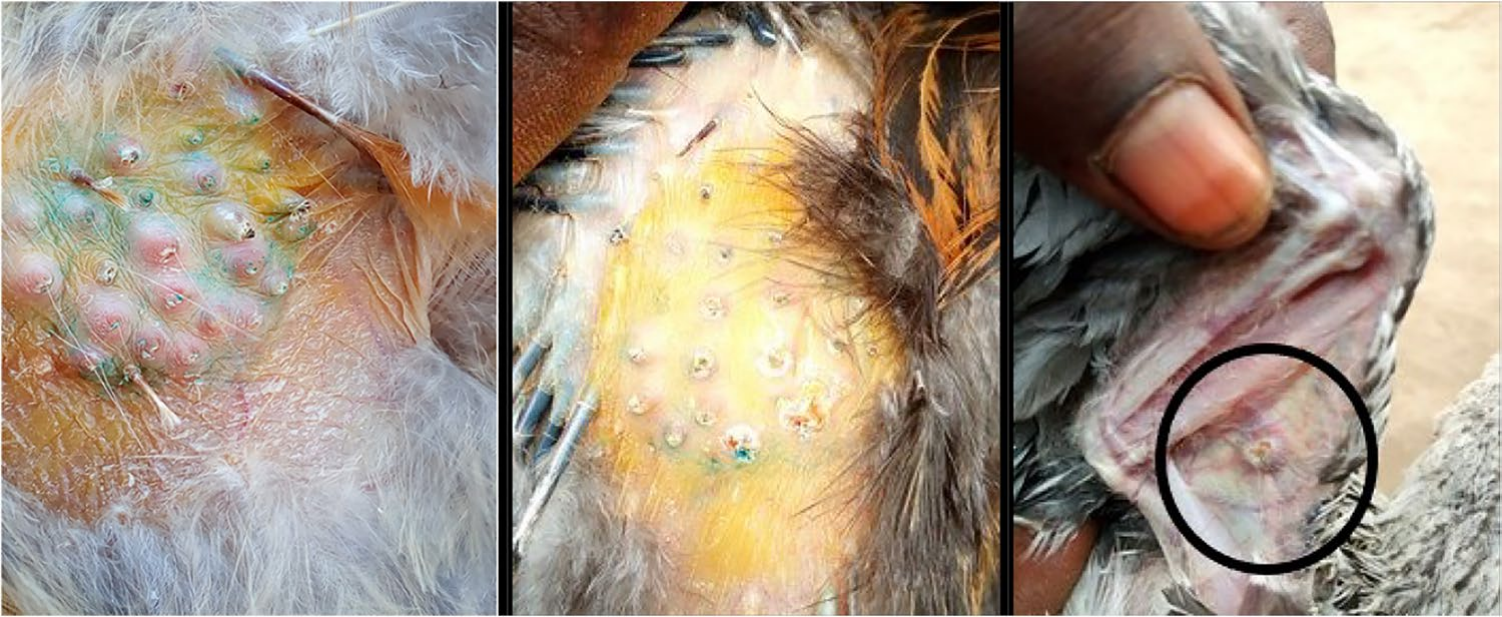
Examples of fowlpox vaccine take reactions on birds 7 or 9 days after vaccination. Nodules on the thigh of a bird (left), scabs on the thigh of a bird (middle) both after vaccination by the feather follicle technique, and a nodule on the wing-web of a bird (right) after vaccination by the stab technique. The presence of such reactions is evidence of successful immunisation

Prior to vaccination on day 0, the administration site (thigh or wing-web) of chickens in groups 1, 3, and 4 were confirmed to be normal. The sites for FP administration were then observed 7 and 9 days after vaccination to assess take reactions and again at the end of the study 21 days after vaccination to assess if take reactions had resolved (Fig. 2). At the first inspection, 7 days after vaccination, 88% (184/208) of the birds in groups 1, 87% (187/214) of the birds in group 3, and 74% (146/196) of the birds in group 3 had a vaccine take reaction. By 9 days after vaccination, take reactions had increased to 96% (190/198) in group 1, 93% (206/222) in group 3, and 96% (194/203) in group 4 (see Fig. 2). By 21 days after vaccination, the take reactions had resolved in all of the birds in groups 1 (184/184) and 4 (164/164), and in 99% (177/179) of birds in group 3.

Overall, the FP vaccine caused the formation of nodules typical of FP in 83% (186/225) of the birds in group 1 and 78% (180/232) of birds in group 3 that were vaccinated by the feather follicle method on the thigh. In comparison, nodules were recorded in 53% (111/210) of the birds that were vaccinated by the wing-web method (group 4). Swelling at the sites of vaccine administration was noted in 76% (170/225) of birds in group 1, 74% (172/232) of birds in group 3, and in 84% (176/210) of birds in group 4. The formation of scabs at the site of vaccination was observed in 69% (155/225) of birds in group 1, 70% (162/232) of birds in group 3, and 78% (164/210) of birds in group 4.

A Bayesian logistic regression model using STAN of the proportion of birds with take reactions in an individual household, with village as a random effect, showed that there was little evidence of overall differences between groups 1, 3 and 4. Non-inferiority analysis showed that neither of the concurrent FP and ND vaccines (groups 3 and 4) was inferior to single FP vaccine (group 1).

#### Serology

**Fig. 3:**
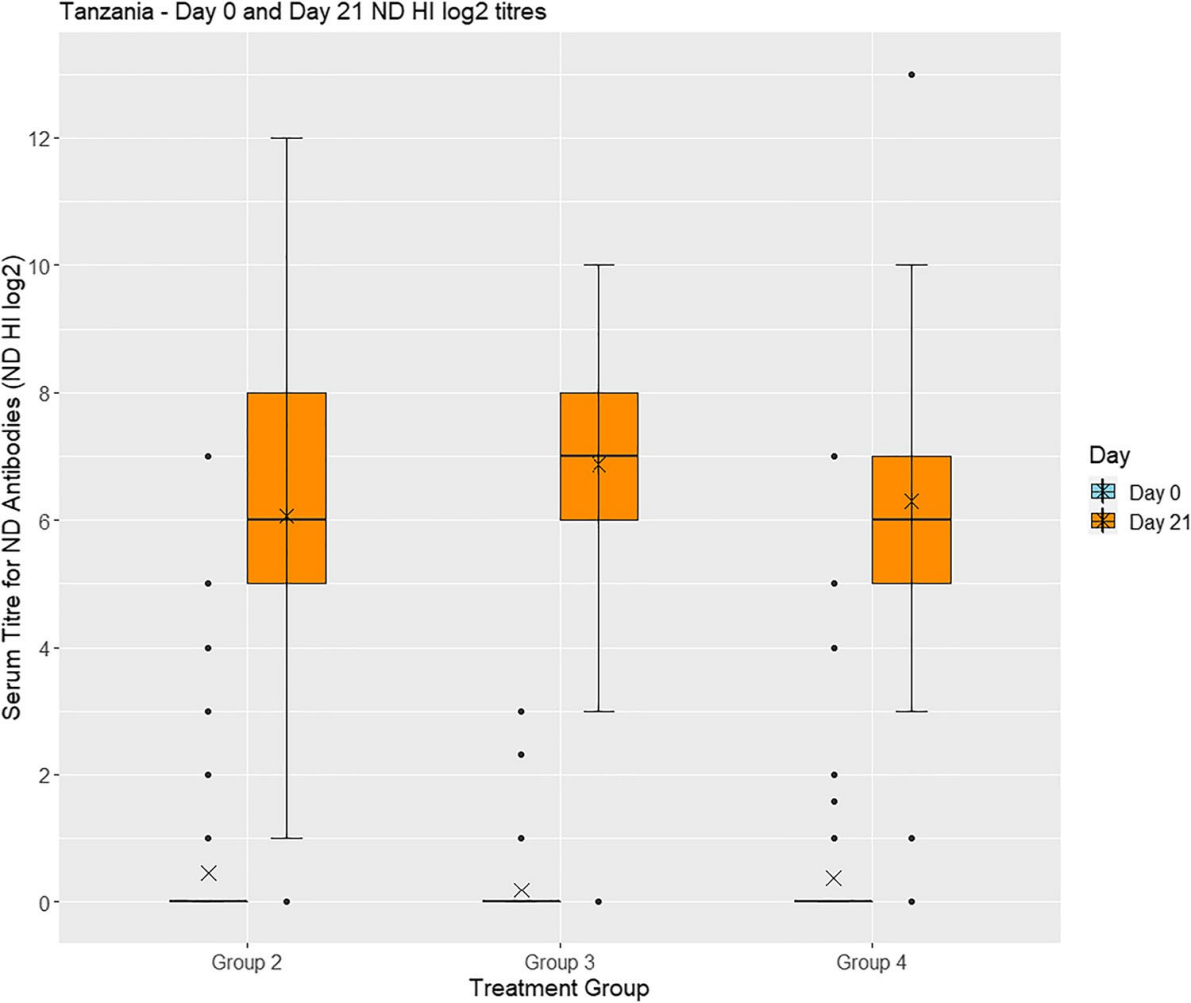
Box plot showing **s**erum ND HI titres in chickens raised in Tanzania. A positive ND HI titre (log_2_ ≥ 4) is evidence of protective immunity to Newcastle disease in chickens. X = mean value. Group 2: vaccinated with ND vaccine by eye-drop; Group 3: vaccinated with FP vaccine (feather follicle technique) and ND vaccine (eye-drop); Group 4: vaccinated with FP vaccine (wing-web stab technique) and ND vaccine (eye-drop)

On day 0, serum samples from birds in groups 2–5 were analysed for ND HI antibodies and the majority found to be negative: 96% (101/105) in group 2, 100% (98/98) in group 3, 97% (83/86) in group 4, and 98% (93/95) in group 5. By 21 days after vaccination, the majority in groups 2–4 were positive, having an antibody titre against ND above the protective level of log_2_ ≥ 4: 86% (42/49) in group 2, 94% (50/53) in group 3, and 90% (37/41) in group 4, while 94% (33/35) of birds in the unvaccinated group 5 were still negative (Fig. 3).

A Bayesian linear regression model using STAN of individual bird log_2_ HI titre values on day 21, with village and household as random effects, showed there was little evidence of overall differences between groups 2, 3, and 4 as shown by the following p-values: 0.422 (group 2 vs. group 3), 0.927 (group 2 vs. group 4), and 0.682 (group 3 vs. group 4). Non-inferiority analysis showed that neither of the concurrent FP and ND vaccines (groups 3 and 4) was inferior to the single ND vaccine (group 2).

### Field study in Nepal

#### Study population

The study population consisted of 51% (532/1037) female and 35% (368/1037) male birds (data for 13% (137/1037) of the birds was not available) with birds of different sex and age being represented in all groups.

#### Mortality

A total of 86 birds died during the study: 26/223 in group 1, 11/186 in group 2, 19/199 in group 3, 10/208 in group 4, and 20/198 in group 5. As with the study in Tanzania, predator attack was given as the main cause of mortality (range 36–70%). No adverse events were reported to be attributable to either ND or FP vaccination.

#### Take reactions

Prior to vaccination on day 0, the administration site (thigh or wing-web) of chickens in groups 1, 3, and 4 were confirmed to be normal. The sites for FP administration were then observed 7 and 11 days after vaccination to assess take reactions and again at the end of the study 28 days after vaccination to assess if take reactions had resolved. At the first inspection 7 days after vaccination, 99% (215/218) of birds in group 1, 99% (192/194) of birds in group 3 and 99% (205/207) birds in group 4 had a take reaction. By 11 days after vaccination, take reactions had increased to 100% (217/217) of birds in group 1, 100% (192/192) of birds in group 3 but decreased slightly to 97% (199/205) in birds in group 4. By 28 days after vaccination, take reactions, mainly seen as swellings, were still visible in 49% (97/196) of the birds in group 1, 48% (87/180) of the birds in group 3, and 37% (73/198) of the birds in group 4. Interestingly, this more detailed analysis showed that at 7 days after vaccination, nodules were the predominant take reaction (86–98%), compared to swellings (20–53%) and scabs (22–51%). By 11 days after vaccination, scabs were the predominant take reaction (85–93%) compared to nodules (16–40%) and swellings (36–59%). By 28 days after vaccination, the predominant take reaction was swellings (34–50%), compared to nodules (9–24%) and scabs (2–11%). A formal statistical analysis of the take reaction data was not possible given that only 1 of the total number of birds vaccinated (620) had never had a take reaction 7 or 11 days after vaccination. Therefore, exact 95% confidence intervals were calculated (98.33–100% for group 1; 98.12–100% for group 3; 97.34–99.99% for group 4) suggesting non-inferiority of combined FP and ND vaccination (groups 3 and 4) compared to single FP vaccination (group 1).

#### Serology

**Fig. 4:**
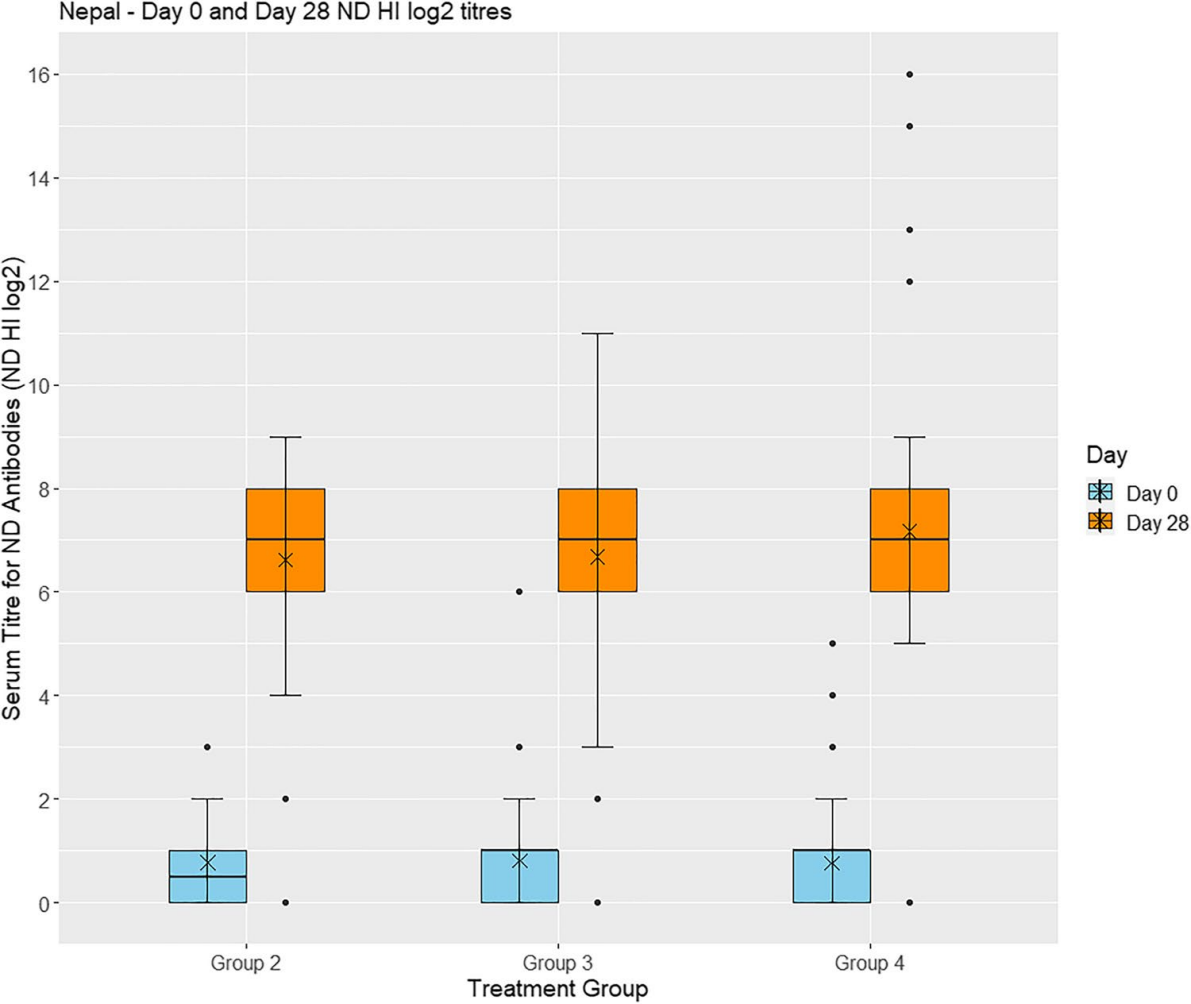
Box plot showing **s**erum ND HI titres in chickens raised in Nepal. A positive ND HI titre (log_2_ ≥ 4) is evidence of protective immunity to Newcastle disease in chickens. X = mean value. Group 2: vaccinated with ND vaccine by eye-drop; Group 3: vaccinated with FP vaccine (feather follicle technique) and ND vaccine (eye-drop); Group 4: vaccinated with FP vaccine (wing-web stab technique) and ND vaccine (eye-drop)

On day 0, serum samples from birds in groups 2–5 were analysed for ND HI antibodies and the majority found to be negative: 100% (162/162) in group 2, 99% (165/166) in group 3, 98% (173/177) in group 4, and 99% (175/176) in group 5. By 28 days after vaccination, the majority in groups 2–4 were positive, having an antibody titre against ND above the protective level of log_2_ ≥ 4: 98% (165/169) in group 2, 97% (166/172) in group 3 and 99% (189/191) in group 4, while 97% (170/176) of birds in the unvaccinated group 5 were still negative (Fig. 4).

A Bayesian linear regression model using STAN of the individual bird log_2_ HI titre values on day 28, with village and household as random effects, showed there was little evidence of overall differences between groups 2, 3, and 4 as shown by the following *p*-values: 0.998 (group 2 vs. group 3), 0.254 (group 2 vs. group 4), and 0.208 (group 3 vs. group 4). Non-inferiority analysis showed that neither of the concurrent FP and ND vaccines (groups 3 and 4) was inferior to the single ND vaccine (group 2). Group 4 showed a higher average titre than group 2 (model estimate difference = 0.294), while group 3 had an almost identical estimate mean log_2_ titre.

## Discussion

Vaccines against fowlpox were among the earliest to be developed for use in poultry (Tripathy and Reed 2008; Giotis and Skinner 2019). Much of this early work started in the 1920s and included the first successful experiments with applying live non-attenuated virus to a scarified area of de-feathered skin or to open feather follicles (Graham and Brandly 1940; Dickinson 1942; Ferguson 1946; Sarma and Sharma 1989; Mockett et al 1990). However, a number of disadvantages to the feather follicle method were identified leading to the so-called stick, or puncture, method of cutaneous vaccination with fowlpox virus in chickens being advocated. Today, the main recommended routes for the administration of fowlpox vaccines in commercial chickens are intramuscular and wing-web stab (OIE 2016). Similarly, the feather follicle method has also been used to vaccinate pigeons against pigeon pox (Woodward and Tudor 1973) although, with the exception of racing pigeons, the wing-web method is also now generally employed in these birds.

GALVmed has been working with partners to develop a vaccination approach for both fowlpox and Newcastle disease that can be easily adopted by trained smallholder farmers, paraprofessionals and community health workers. This has led to the return to the older method of applying fowlpox vaccine via the feather follicles (Stuke et al 2020). A POC study had previously proven the safety and efficacy of the concurrent administration of live fowlpox and Newcastle disease vaccines via both non-invasive routes: feather follicle for fowlpox, eye-drop for Newcastle disease (Stuke et al 2017). The objective of these two field studies was to confirm the safety and efficacy of this approach in rural, free-range, scavenging, indigenous chicken representative of the local populations in Tanzania and Nepal.

The demonstration of low levels of antibodies in most of the birds before vaccination (3% in Tanzania, 1% in Nepal) indicates that neither population of birds had been recently exposed to Newcastle disease or that they may have had residual amounts of maternally derived antibodies. None of the households in either country were reported to have undertaken Newcastle disease vaccination during the previous four months. Similarly, both bird populations were also naïve to fowlpox as almost all vaccinated birds developed take reactions. That these populations of birds were naïve to both Newcastle disease and fowlpox highlights the importance of regular vaccination to avoid disease outbreaks. In recent years, outbreaks of fowlpox have been gradually increasing mostly due to the emergence of novel strains of the virus (the virulence of which is reportedly enhanced by the integration of reticuloendotheliosis virus into the fowlpox viral genome (Umar et al 2021)), especially in tropical and sub-tropical regions of the world, where the control of biting insects is less efficient (Giotis and Skinner 2019; Umar et al 2021) resulting in economic loss for both large and small-scale poultry farmers (Abdu and Musa 2014). On the completion of both field studies, almost all vaccinated birds were positive to Newcastle disease (91% in Tanzania, 98% in Nepal). That a proportion of the Newcastle disease vaccinated birds (6–14% in Tanzania, 1–4% in Nepal) had antibodies levels lower than the protective threshold of log_2_ ≥ 4 3 to 4 weeks after vaccination was not surprising and has been reported before. For example, between 2 and 16% of scavenging village chickens in Malawi failed to produce a protective antibody response to Newcastle disease 4 weeks after vaccination with I2 Newcastle disease vaccine by eye-drop (Mgomezula et al 2005). Studies have also shown that lower Newcastle disease HI titres after vaccination in extensively managed chickens, as compared with those reared in intensive production systems, can be a result of factors such as concurrent infection with parasites (Abera et al 2017) as well as poor nutrition, stress, and the physical environment adversely effecting immune responses. Although a number of the unvaccinated birds had positive HI titres to Newcastle disease at the end of the studies (6% in Tanzania, 3% in Nepal), overall, the antibody titres in this group did not increase greatly (0.4 to 1.1, and 0.7 to 0.8 on the log_2_ scale, respectively, for Tanzania and Nepal) indicating that most of the flocks in the study areas were not exposed to Newcastle disease during the duration of the studies.

Between 94–96% (Tanzania) and 99–100% (Nepal) of the non-invasive, concurrently vaccinated birds had take reactions, which is comparable to the 70–95% of birds with take reactions seen when reared under controlled laboratory conditions in the GALVmed POC study (Stuke et al 2017). When categorised into nodules, swellings, and scabs, a trend was observed in birds in Nepal with predominantly nodules being observed in 92% of birds across all groups about one week after vaccination, followed by the formation of scabs, a final stage in the healing process, in 88% of birds across all groups at eleven days after vaccination. A similar trend has been reported before (Broerman and Edgington 1932; Graham and Barger 1935; Alehegn et al 2014). The product literature for live fowlpox vaccines generally states that take reactions will usually resolve within two to three weeks after wing-web application. A similar time frame has been reported in the literature for live fowlpox vaccines administered by the feather follicle method and was also noted in the POC study with only 1/20 birds having a small lesion 19 days after vaccination, and only 2/527 birds having a positive take reaction 21 days after vaccination in the current field study from Tanzania. It was therefore interesting to note that in the study in Nepal, take reactions — the assessment of which can be a subjective process — were still visible in 45% of the vaccinated birds 28 days after vaccination: 15% with nodules, 42% with swellings, and 5% with scabs. That it might take more than 3 weeks after vaccination for take reactions to fully resolve has been reported by others (Graham and Barger 1935; Gerlach 1994; Alehegn et al 2014). However, it would appear that the take reactions had in fact almost resolved by 11 days after vaccination with between 85 and 93% of all vaccinated birds having scabs, most of which were subsequently reported to have dropped off by about four weeks after vaccination. In addition, there were no records of any of the birds becoming ill or having reduced feed consumption during the last couple of weeks of the study which would tend to rule out any significant bacterial or fungal secondary infections complicating the healing process.

No adverse events were attributed to vaccination in either field study, with levels of mortality comparable to those reported from other areas with village production systems being characterised by minimal human intervention, birds scavenging for food and predation often being a major cause of mortality (Selam and Kelay 2013), although Holmern and Røskaft (2014) highlight that calculating losses due to predators can be complex and is not always straightforward.

In conclusion, both field studies demonstrated that the concurrent administration of commercial live attenuated fowlpox and Newcastle disease vaccines by non-invasive routes is safe and induces levels of protective immunity against fowlpox and Newcastle disease that are non-inferior to fowlpox or Newcastle disease vaccinations alone. There was also no interference between the two live vaccines. Feedback from some of the participants in Tanzania and Nepal was also supportive of such a vaccination approach. Overall, the application of the fowlpox vaccine via the feather follicle method was perceived as an easy technique and that observing the presence of take reactions provided reassurance that vaccination had worked. The brush applicators could be made locally using wood splinters and cotton wool; even commercial human ear brushes could be used. In areas where there is a limited availability of trained paraprofessionals and community health workers many farmers felt confident of being able to administer fowlpox vaccine via the feather follicle method following some basic training. The co-administration of both fowlpox and Newcastle disease vaccines by non-invasive routes therefore appears to be beneficial in terms of increasing the uptake of such vaccines by trained small-scale backyard poultry farmers thus helping to control both of these diseases.

## Data Availability

The authors declare that all datasets generated during the two studies will be made available in a freely accessible online data repository.
